# Spinal Cord Infarction: Access Site Complication of Femoral Artery Percutaneous Coronary Intervention

**DOI:** 10.7759/cureus.60666

**Published:** 2024-05-20

**Authors:** Lubna Saffarini, Rand Aboelkher, Nour Sabobeh, Talha Abdelfattah, Mahmoud Naji

**Affiliations:** 1 Emergency Medicine, Rashid Hospital, Dubai, ARE

**Keywords:** spinal cord syndrome, spinal cord ischemia, spinal cord infarction, bilateral lower limb weakness, access site complication, primary percutaneous coronary intervention (pci)

## Abstract

Spinal cord infarction (SCI) is an uncommon vascular syndrome that leads to neurologic abnormalities with multiple implicated causes. Percutaneous coronary intervention (PCI) is a non-surgical invasive procedure used to relieve an arterial occlusion or narrowing that causes ischemia to the heart. This is usually performed by different methods and different arterial access sites. Here, we present a case of a patient who developed bilateral lower limb weakness eight days after a femoral artery PCI and was diagnosed with SCI. This case report aims to document a rare complication and highlight the most important demographic, investigation, management, risk factors, and prognosis data available in the literature.

## Introduction

Spinal cord infarction (SCI) is an uncommon disease syndrome, and its incidence is poorly documented in the literature [1]. One study noted that it accounted for 1.2% of all strokes while another paper stated that the syndrome accounts for 0.3% to 1% of all strokes [2]. It was recently observed that SCI accounts for 1% to 2% of ischemic strokes and 5% to 8% of all acute myelopathies [3].

SCI is relatively uncommon due to the spinal cord’s extensive and intricate network of collateral vascular supply [3]. The spinal cord’s primary blood supply comes from the single anterior spinal artery (ASA) and the two posterior spinal arteries (PSA) [4]. The ASA forms from the vertebral arteries that come from the first part of the subclavian artery [4]. The ASA is responsible for supplying blood to the anterior part of the spinal cord, and it also gives rise to the sulcocommissural artery which also supplies the anterior part [3]. In supplying the ASA, around five to eight of the radicular arteries play a dominant role, with one of the thoracolumbar arteries originating from T9 to T12 taking that role in 90% of people [4]. This artery of Adamkiewicz provides perfusion to the lower thoracic and lumbar spinal cord, as well as the conus medullaris [4]. The lower thoracic spinal cord is at greater risk of infarction due to its hypovascularity and lower degree of collateral circulation [4]. On the other hand, PSA can arise from the posterior inferior cerebellar arteries (PICA), but it also depends on posterior radicular arteries, which come from a vertebral artery, for its blood supply [4]. The PSA supplies the posterior columns, posterior dorsal horns, and portions of the corticospinal and spinothalamic tracts [4].

Itakagi et al. hypothesize that SCI is believed to occur after a PCI procedure due to the disturbances of blood flow caused by a balloon catheter entering the femoral artery to the common iliac artery and aorta [5]. This results in perfusion pressure reduction of the internal iliac artery, which then reduces blood supply to the spinal cord [5]. Another hypothesis is that the catheter advances in an artery with progressing arteriosclerosis, which causes a plaque rupture, an embolism to develop, and therefore ischemia [5]. This is further supported by Keeley and Grines who studied 1000 patients and found that more than 50% of PCI procedures resulted in atherosclerotic plaque scraping and dislodging, however, they noted it was not associated with complications during the patient’s hospital stay [6].

In this case report, we will describe an incident where a patient developed SCI as an access site complication (ASC) of femoral artery PCI.

## Case presentation

A 61-year-old male with type II diabetes mellitus (DM) and ischemic heart disease (IHD), presented to our emergency department (ED) with a sudden onset of numbness and weakness in both legs. His symptoms began eight hours before attending the emergency with numbness in his lower limbs which then progressed to involve his abdomen, finally leading to weakness in both legs and inability to urinate or pass bowel movements. He denied any previous similar episodes, recent trauma, or back pain, and the review of systems was unremarkable for fever, cough, night sweats, weight loss, insect bite, recent travel, or sick contacts.

On further history, he reported having a recent myocardial infarction eight days ago which was treated in a different hospital. His transport via ambulance was complicated by an out-of-hospital cardiac arrest that lasted for a brief moment and reverted immediately with the first defibrillation attempt. He was received in a facility where he underwent primary PCI with a right femoral artery access site. His coronary angiography (CAG) reported total occlusion in the proximal left anterior descending (LAD) artery with a thrombotic lesion, managed by the insertion of one drug-eluting stent (DES). Upon discharge, he was started on multiple medications including aspirin 100 mg, once daily, ticagrelor 90 mg twice daily, atorvastatin 80 mg once daily, bisoprolol 2.5 mg once daily, empagliflozin 10 mg once daily, eplerenone 25 mg once daily, and pantoprazole 40 mg once daily.

On initial evaluation, his vital signs were within normal ranges and he was oriented to time, place, and person. Cardiopulmonary examinations were unremarkable. He was noted to have bruises over the right groin on exposure, and his abdomen was soft on physical exam with suprapubic tenderness. His neurological examination was significant for lower extremities weakness (Table 1), a sensory deficit from the lower limbs up to the sensory level of D7, a non-sustained clonus in the upper limbs, an inability to walk, and a weak anal sphincter tone.

**Table 1:** Motor examination of specific muscle groups in the upper and lower limbs. Power score is defined as 0 for no muscle activation or movement, 1 for minimal muscle activation (example: twitching) without achieving full range of motion, 2 for muscle activation with gravity eliminated and achieving full range of motion, 3 for muscle activation against gravity achieving a full range of motion, 4 for muscle activation against some resistance achieving a full range of motion, and 5 for muscle activation against an examiner’s full resistance achieving a full range of motion.

Upper and lower limb muscle groups examined for power	Power score
Right arm abduction	5/5
Right arm adduction	5/5
Right arm flexion	5/5
Right arm extension	5/5
Right fingers flexion	5/5
Right hip flexion	0/5
Right knee extension	0/5
Right dorsiflexion	0/5
Right plantar flexion	0/5
Left arm abduction	5/5
Left arm adduction	5/5
Left arm flexion	5/5
Left arm extension	5/5
Left fingers flexion	5/5
Left hip flexion	0/5
Left knee extension	0/5
Left dorsiflexion	0/5
Left plantar flexion	0/5

His cranial nerve examination was unremarkable (Table 2). Rectal examination elicited reduced anal tone. Bedside point of care ultrasound (POCUS) revealed a distended urinary bladder, hence a foley catheter was inserted, and the procedure was done without difficulties with an initial urine drain of 1000 mL of urine.

**Table 2:** Detailed examination findings of the cranial nerves. CN: Cranial nerves.

Cranial nerve	Examination finding
CN II	Pupils equal and reactive, no visual field deficits
CN III, IV, VI	Intact extraocular muscle movements, no gaze preference or deviation, no nystagmus
CN V	Normal sensation in V1, V2, and V3 segments bilaterally
CN VII	No asymmetry, no nasolabial fold flattening
CN VIII	Normal hearing to speech
CN IX, X	Normal palatal elevation, no uvular deviation
CN XI	5/5 head turn and 5/5 shoulder shrug bilaterally
CN XII	Midline tongue protrusion

Laboratory workup revealed leukocytosis (WBC of 18.2 103 uL) with neutrophilic prominence (PMN percentage was 85.1%), thrombocytosis (platelets count of 622 103 uL), hyperkalemia (K of 5.5 mmol/L), hyponatremia (Na 135 mmol/L), uremia (urea of 61 mg/dL), deranged kidney function (Cr 1.73 mg/dL, estimated glomerular filtration rate (eGFR) 44.4 ml/min/1.73 m2), and mildly elevated inflammatory markers (CRP 38.1 mg/L, ESR 72 mm/1hr, procalcitonin 0.14 ng/mL). Pan-cultures collected on presentation revealed no growth of any organisms.

**Figure 1:**
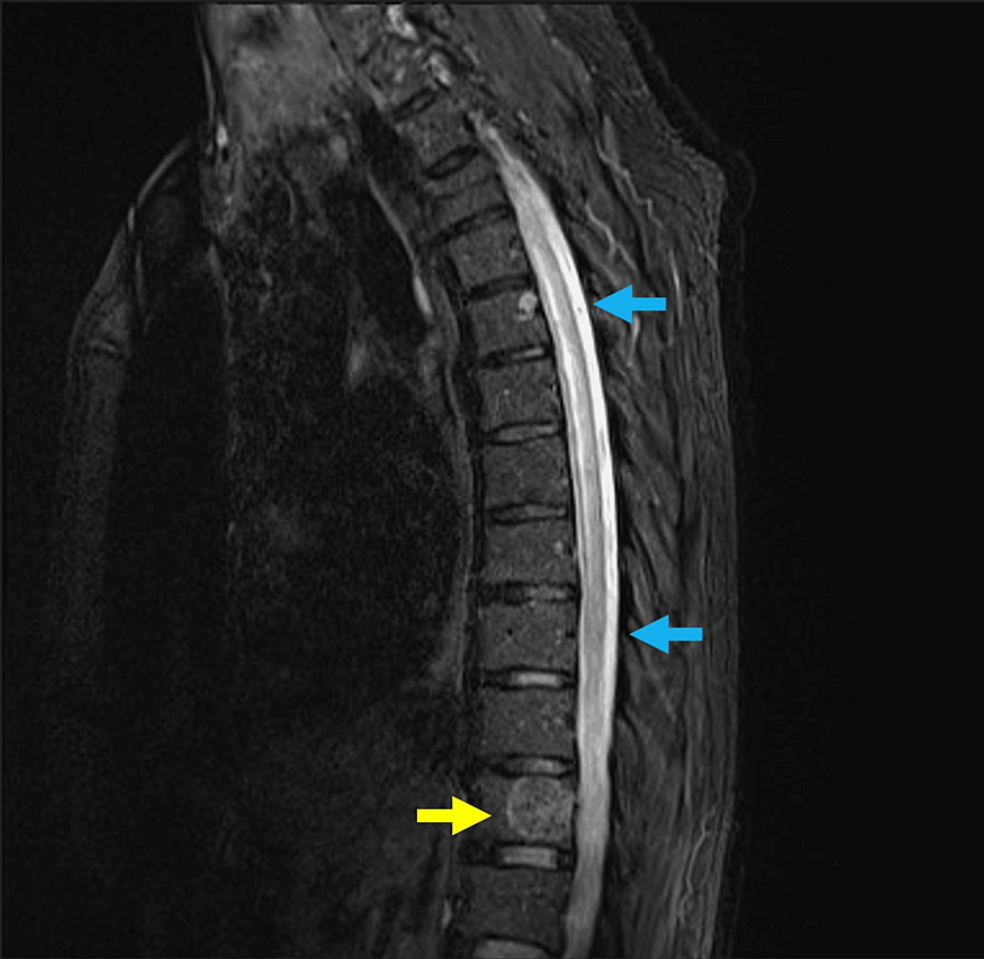
Increased intramedullary spinal cord signals with a vertebral body hemangioma. Sagittal T2 view showing increased intramedullary spinal cord signals predominantly involving the central grey matter from D4-D11 (blue arrows) with an incidental finding of vertebral body haemangioma (yellow arrow).

**Figure 2:**
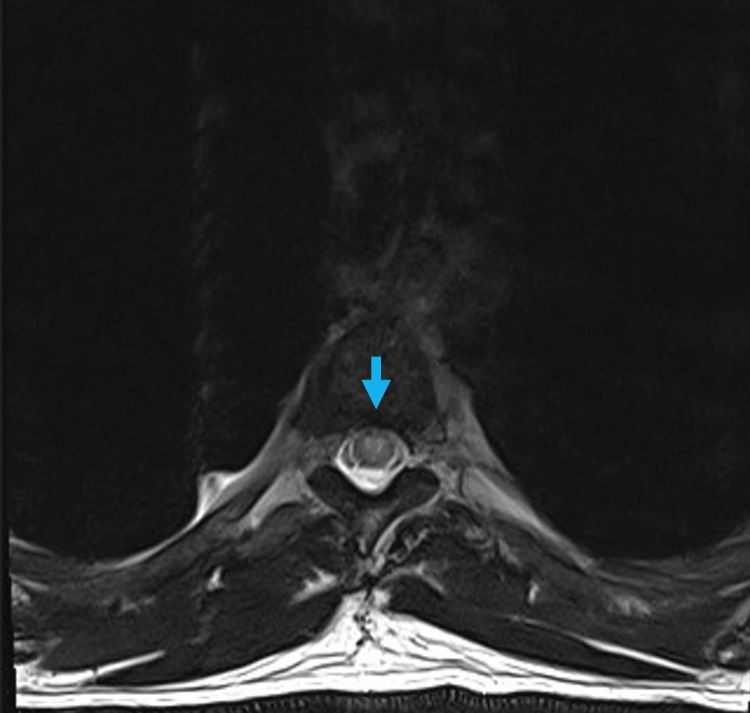
Owl’s eye appearance. Axial T2 image showing an intramedullary increased signal resembling an owl’s eye (blue arrow).

A cervical and thoracic magnetic resonance imaging (MRI) with contrast was done. The sagittal T2 view showed increased intramedullary spinal cord signals predominantly involving the central grey matter from D4-D11, with an incidental finding of vertebral body haemangioma (Figure 1). The axial T2 image showed an intra-medullary increased signal resembling an owl’s eye appearance, with no significant cord expansion (Figure 2). These findings are consistent with the diagnosis of an SCI.

The patient was started on intravenous (IV) methylprednisolone one gram daily which was continued for five days, empirical IV ceftriaxone 2 grams daily for five days, gastrointestinal (GI) and deep vein thrombosis (DVT) prophylaxis, along with patient’s home medications. Further investigations including a vasculitis workup and a pan-CT to rule out para-neoplastic syndrome turned out to be unremarkable. Transthoracic echocardiogram (TTE) showed normal left ventricular (LV) cavity dimensions with adequate LV systolic function, LV ejection fraction of 50% to 56%, trivial mitral regurgitation (MR), trivial aortic regurgitation (AR), trivial tricuspid regurgitation (TR), and a thin rim of pericardial effusion.

During his stay, his symptoms continued to progress resulting in total paresthesia of the lower extremities up to the level of D7, and complete paralysis of the lower extremities with a power of 0/5 bilaterally. He eventually requested to go back to his home country for further evaluation and treatment and was discharged on day 10 of admission.

## Discussion

The occurrence of SCI as an ASC of femoral artery PCI is even less documented in the literature. Despite a thorough literature review, we noticed that mainly case reports described the occurrence of SCI as a complication of PCI. We found four case reports [7-10]; one was in Spanish with an English translation of the abstract only [10], so data was collected from there for writing this review. One case was described and presented as a poster at the 2020 Annual Meeting of the American Academy of Neurology (Abstract: Sista SR, Nersesyan H. Ischemic Myelopathy as a Perioperative Complication of Percutaneous Coronary Intervention via Femoral Approach; 29/04/2020; https://www.aan.com/MSA/Public/Events/AbstractDetails/44696). The American Journal of Medicine also described two other cases in the “Brief Clinical Observations” section of volume 87 [11]. Cumulatively, the cases were published in 2020, 2014, 2009, 2000, 1996, and 1989 [7-11] (Abstract: Sista SR, 29/04/2020). With a total of seven patients, 57% were male with a median age of 66 years (range: 47-80 years). The access site was the femoral artery in three cases (42.9%) [8,9] (Abstract: Sista SR, 29/04/2020) whereas in all other patients, it was not specified [7,10,11]. Four patients (57.1%) developed low back pain accompanied by bilateral lower limb weakness and decreased sensation [7,9,10,11], where 50% of them had decreased tendon reflexes [7,9], and one patient had reduced anal sphincter control [10]. Three out of seven patients (42.9%) didn’t complain of back pain [8,11] (Abstract: Sista SR, 29/04/2020), yet two of them had lower limb weakness and paresthesia bilaterally [8,11]. One patient, however, had only right lower limb weakness (Abstract: Sista SR, 29/04/2020). The symptoms developed immediately after the procedure in five patients [7-10,11], the other two patients developed their symptoms within their first postoperative day [11] (Abstract: Sista SR, 29/04/2020).

The initial study of choice for diagnosing an SCI is an MRI. Although it is not sensitive enough to detect it acutely, it is, however, essential to exclude other diagnoses, mainly spinal cord compression syndromes [12]. Similarly, diffusion-weighted imaging demonstrating a T2 hyperintensity can be used even though it also has a low sensitivity [12]. In the documented seven cases of SCI in the literature, six of them (85.7%) underwent a diagnostic MRI [7-11] (Abstract: Sista SR, 29/04/2020), and T2 hyperintense signals in the spinal cord were seen in four out of the six patients (66.6%) [7-9] (Abstract: Sista SR, 29/04/2020) which corresponded to their clinical presentation. The MRI findings were not documented for one patient [10], whereas it was normal in another patient and a clinical diagnosis of SCI was made [11]. One patient (14.3%) underwent emergency myelography and aortography [11], which were both normal, and the diagnosis of SCI was done clinically. As per Nasr and Rabinstein, if an MRI is not available or is contraindicated then a CT myelography can be used [12].

There is no consensus guideline published regarding the management of SCI and it is not sufficiently studied [12]. A few case reports advocate for IV thrombolysis in the hyperacute state, lumbar drains have been suggested for patients with SCI after the repair of a thoracoabdominal aortic aneurysm, and steroids are generally not recommended except for patients with evidence of vasculitis [12]. Otherwise, treatment usually includes control of risk factors, antithrombotic or anticoagulation therapy, and pain control [12]. In the cases we’ve extrapolated from the available literature, the management was not mentioned for four out of seven patients (57.1%) [10,11] (Abstract: Sista SR, 29/04/2020). One patient received steroids IV for a total of five days along with aspirin, enoxaparin, and physiotherapy, and he showed evidence of improvement after a two-month follow-up in his power, voluntary anal contraction, and reduction in infarct size on a repeat MRI [8]. On the other hand, another patient only received physiotherapy in a rehabilitation hospital and he never regained ambulation [7], and the third patient was anticoagulated while also being in a rehabilitation program and he showed improvement in his two-month follow-up [9]. The four patients with an undocumented management plan were also followed up at different time intervals [10,11] (Abstract: Sista SR, 29/04/2020). One patient’s neurological status was unchanged at the six-month follow-up [10], and two patients died during their hospital stay, on day 57 and day 32 respectively [11]. It was noted that their symptoms did not improve before their passing [11]. One patient’s prognosis was not documented (Abstract: Sista SR, 29/04/2020). Similar to the short-term outcomes described in the previous patients, there are limited studies describing the long-term outcomes of SCI [13].

Romi and Neiss also stated that cardiac diseases are less common among SCI patients despite common risk factors (for example diabetes mellitus (DM), peripheral arterial disease, smoking, and cholesterol) [13]. Thus, they concluded that those risk factors contribute to atherosclerosis in both pathologies and hence why SCI is not associated directly with cardiac disease [13]. They also noted that the presence of hypertension and elevated blood glucose levels on admission regardless of DM is associated with more severe spinal cord strokes [13]. In the seven patients reviewed, risk factors weren’t documented for all cases. Ischemic heart disease and cardiomyopathy were described in two patients, one of them was able to walk on crutches while the other had an unchanged course at the six-month follow-up [9,10]. One patient was documented to have DM, hypertension, and hypercholesterolemia and he never regained ambulation [7].

In comparison to the case reports published to our knowledge regarding the topic of SCI as a complication of femoral artery PCI, our patient developed the syndrome after eight days from his femoral artery PCI; unlike other documented cases where it developed immediately after the procedure or within the first day. Moreover, most patients complained of back pain with bilateral lower limb weakness and paresthesia whereas our patient denied any history of back pain. Despite the controversy in management, our patient received a five-day course of IV steroids even though later his vasculitis workup turned out to be negative. He also received aspirin, enoxaparin, and daily physiotherapy which didn’t improve his deficit before discharge on day 10. Unfortunately, we don’t have any contact with our patient to ask about his symptoms. Even though another patient similarly received the same management as our patient, he had improvement in his two-month follow-up [8]. One more patient improved after a two-month follow-up, but he only received anticoagulation and was enrolled in a rehabilitation program without the prescription of steroids [9]. Our patient had DM and IHD, his blood sugar levels were maintained throughout his hospital stay and his vitals were stable.

## Conclusions

The occurrence of spinal cord infarction (SCI) as an access site complication of femoral artery percutaneous coronary intervention (PCI) is not well recognized in the literature, which raises the question of whether it is due to a lack of documentation or a rarity of occurrence. The exact mechanism and pathophysiology are still being investigated, but it is believed to occur due to blood flow disturbances or following the rupture of an atherosclerotic plaque. Symptoms of SCI include back pain, lower limb weakness, lower limb paresthesia, urine retention, and loss of anal sphincter control depending on the level being affected. The initial study of choice is an MRI even though it is not sensitive enough and there is no unanimity in deciding a management plan as there are no consensus guidelines published. Despite a thorough literature review, we only found four case reports, two cases documented in a medical journal, and one case presented as a poster at a conference describing the occurrence of SCI as a complication of PCI. More research is needed to better understand the risk factors, mechanisms, and treatment of SCI as a complication of PCI.
